# Diagnosis and multimodal therapy for extragastrointestinal stromal tumor of the prostate: A case report

**DOI:** 10.3892/etm.2013.1156

**Published:** 2013-06-14

**Authors:** ZHENYU OU, ZHENZHEN CAO, YAO HE, DIHONG TANG

**Affiliations:** 1 Department of Urology, Xiangya Hospital, Central South University, Changsha, Hunan 410008;; 2 Department of Gynecologic Oncology, The Affiliated Tumor Hospital of Xiangya Medical School, Central South University, Changsha, Hunan 410013, P.R. China

**Keywords:** extragastrointestinal stromal tumors, prostate, diagnosis, targeted therapy

## Abstract

Extragastrointestinal stromal tumors (EGISTs), which are neoplasms outside the digestive tract, are predominantly observed in the greater omentum and retroperitoneum. The clinicopathological and molecular characteristics of EGISTs are similar to those of gastrointestinal stromal tumors (GISTs). EGISTs originating from the prostate are extremely rare. In this study, we report a case of a prostatic EGIST in a 39-year-old male, who presented with frequency, urgency, dysuria and a prostatic mass. A 10-core transrectal ultrasound-guided prostate biopsy was performed, and the histological and immunohistochemical results confirmed the diagnosis of EGIST. The patient received a radical prostatectomy, followed by targeted therapy with imatinib (400 mg, daily) for 1 year. Neither recurrence nor metastasis was detected at a 24-month follow-up.

## Introduction

Gastrointestinal stromal tumors (GISTs) are the most common form of mesenchymoma originating from the gastrointestinal tract. In these GISTs, approximately 70% originate from stomach, 20–30% from the small intestine and less than 10% from the colon, rectum and esophagus. It is thought that GISTs arise from the interstitial cells of Cajal, and predominantly express immunoreactivity for CD117, a tyrosine kinase growth factor receptor (1). Extragastrointestinal stromal tumors (EGISTs), which exhibit similar clinicopathological and immunohistochemical features to GISTs, are primarily observed in the greater omentum, mesentery and retroperitoneum (2). Immunohistochemical studies aid the correct diagnosis of EGIST. More than 95% of EGISTs express CD117 and 50–100% express CD34, while few are stained positively for desmin, S-100 protein, or smooth muscle actin. Primary EGISTs originating from the prostate are rarely encountered. To the best of our knowledge, only sporadic cases of a confirmed primary EGIST of the prostate have been recorded (3,4). In this study, we present a rare case of a primary EGIST of the prostate, and describe its treatment. To the best of our knowledge, this may have been the first case of a prostatic EGIST treated using multimodal therapy, including radical prostatectomy and imatinib.

## Case report

**Figure 1.**
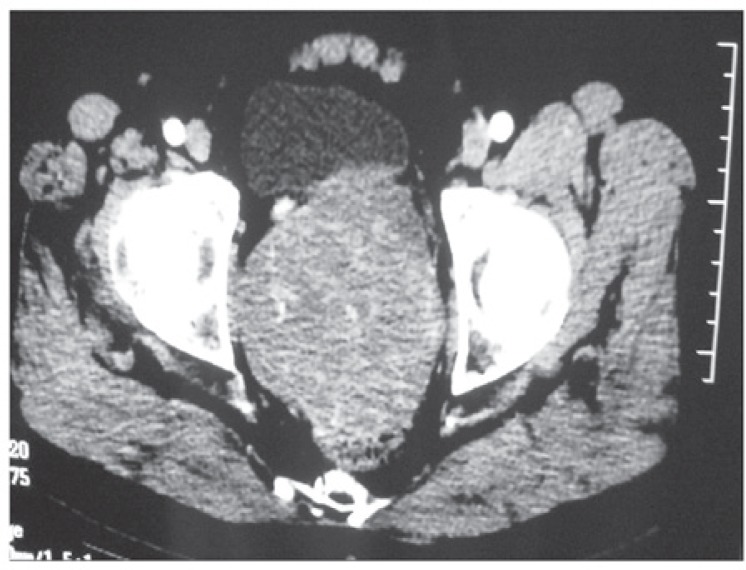
Pelvic computed tomography (CT) scan revealing a heterogenous mass occupying the majority of the prostate.

**Figure 2.**
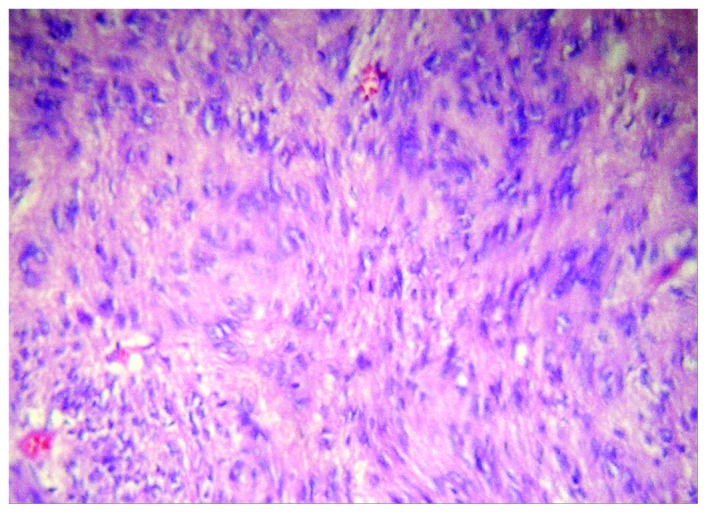
Composition of the tumor. Light microscopy revealed that the tumor primarily consisted of spindle cells (hematoxylin and eosin staining; magnification, ×200).

**Figure 3.**
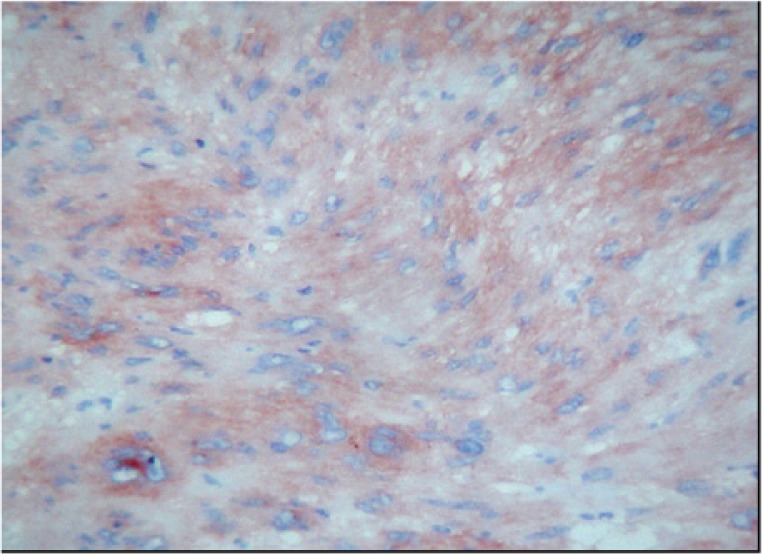
Immunohistochemical analysis of the tumor cells. Results demonstrate that the tumor cells were positive for CD117 (immunohistochemical staining; magnification, ×200).

**Figure 4.**
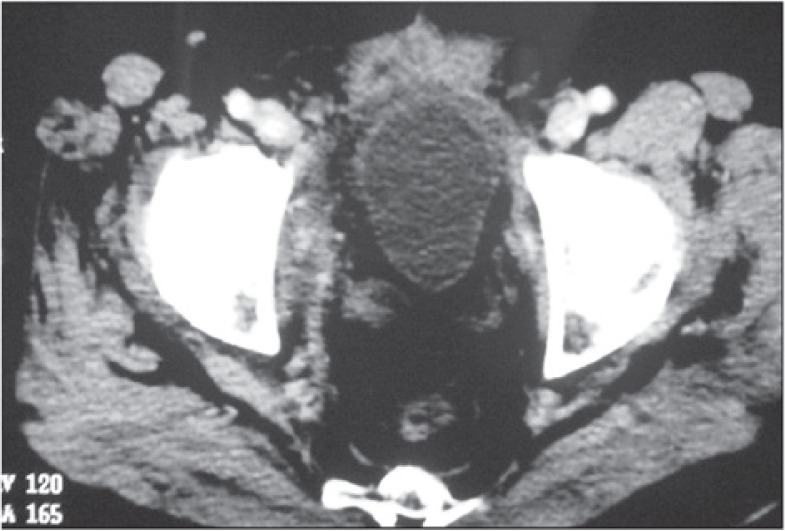
Computed tomography (CT) scan exhibiting no evidence of recurrence, 24 months following the surgery.

A 39-year-old male presented with frequency, urgency and dysuria for 1 year. A digital rectal examination demonstrated a markedly enlarged prostate that exhibited the usual consistency, and tenderness on palpation. The serum level of prostate specific antigen (PSA) was 0.87 ng/ml, and the carcinoembryonic antigen (CEA) and carbohydrate antigen (CA) 19-9 levels were normal. Pelvic computed tomography (CT) revealed an enlarged prostate (10×6 cm) that compressed the rectum and urinary bladder, and that appeared heterogeneous, following contrast enhancement (Fig. 1). Enteroscopy revealed no abnormalities, and no metastases were identified in any other organs through ultrasonography or CT scans. A 10-core transrectal ultrasound-guided prostate biopsy was performed, in order to obtain pathological specimens. The light microscopy examination demonstrated that the tumor primarily consisted of spindle cells (Fig. 2). The immunohistochemical analysis was positive for CD117 (Fig. 3), CD34 and vimentin, while negative results were obtained for cytokeratin, desmin, S100 protein and smooth muscle actin. A pathological diagnosis of EGIST of the prostate was made, prior to the patient undergoing a radical prostatectomy. Intraoperatively, the tumor was noted to be confined to the prostate, without the involvement of the rectum, and no enlarged pelvic lymph nodes were detected. The size of the resected prostate specimen was 10.0×7.0×6.5 cm, and the final pathological examination confirmed the diagnosis of prostatic EGIST. The surgical margins were evaluated, and a positive microscopic margin was identified at the tip of the prostatic apex. Subsequently, the patient received targeted imatinib therapy (400 mg, daily) for 1 year. The patient was placed under observation for 24 months, and, in that period, no recurrence or metastasis was exhibited (Fig. 4). This case report was approved by the ethics committee of the Central South University (Changsha, China), and informed patient consent was obtained.

## Discussion

Neoplasms that occur as primary tumors outside the alimentary tract, and that exhibit similar morphological, immunophenotypical and molecular genetic characteristics to GISTs, are known as EGISTs. Due to the EGISTs, it is important to confirm whether the tumor is associated with the digestive tract, prior to making the diagnosis of EGIST. There are few case reports concerning EGISTs with a potential origin in the prostate; however, several cases of GISTs arising from the rectum have been misdiagnosed as prostatic EGISTs (5). When making a diagnosis of prostatic EGIST, it is necessary for clinicians to be prudent. The first case of a GIST that potentially originated from the prostate was revealed by Van der Aa *et al* (5). A 49-year-old male was demonstrated to have a large prostatic mass, and a biopsy revealed the presence of a GIST. Treatment with imatinib resulted in a reduction in the size of the mass. However, it was not possible to confirm the diagnosis of prostatic EGIST in the absence of surgical excision. In the present case, enteroscopy and CT revealed no abnormalities intra- or extrarectally, and the light microscopy examination and immunohistochemical analysis of the biopsy specimens confirmed the pathological diagnosis of GIST. Intraoperatively, the tumor was noted to be confined to the prostate, without involvement of the rectum. The preoperative examinations, pathological results and intraoperative investigations confirmed that the tumor was an EGIST, primarily originating from the prostate. Two additional cases of GISTs of a prostatic origin have been studied by Lee *et al* and Yinghao *et al* (3,4). The patients concerned in these cases received radical prostatectomy, which revealed that the tumors were confined to the prostate.

Surgical resection is currently the primary treatment option for non-metastatic EGISTs (6). For prostatic masses, transrectal ultrasound-guided prostate biopsies may assist in the determination of a treatment strategy. With regard to prostatic EGISTs, radical prostatectomy is considered to provide satisfactory results. In the present case report, a radical prostatectomy was conducted on the patient, since preoperative examinations did not reveal metastases. Imatinib, a selective protein tyrosine kinase inhibitor, has been demonstrated to be an effective treatment for GISTs and EGISTs (7–10). In the present case, the postoperative pathological examination revealed a positive microscopic margin in the surgical specimen. As a result of this, the patient was considered to be at a high risk of recurrence; therefore, the patient received imatinib treatment for 1 year, in addition to surgery. In the two cases of prostatic EGISTs mentioned previously, the patients received surgical treatment without imatinib therapy, since positive microscopic margins were not identified. However, in the present case, the absence of recurrence or metastasis in the 24-month follow-up period indicated that surgery combined with imatinib therapy was an effective course of treatment for this patient.

In conclusion, this study presents a rare case of an EGIST originating from the prostate, which was treated using multi-modal therapy. The results from this case indicate that surgical resection, followed by imatinib therapy, may offer a promising outcome for patients diagnosed with prostatic EGIST, where there is a high risk of recurrence.
